# Preclinical Testing of New Hydrogel Materials for Cartilage Repair: Overcoming Fixation Issues in a Large Animal Model

**DOI:** 10.1155/2021/5583815

**Published:** 2021-06-19

**Authors:** Benedict Lotz, Friederike Bothe, Anne-Kathrin Deubel, Eliane Hesse, Yvonne Renz, Carsten Werner, Simone Schäfer, Thomas Böck, Jürgen Groll, Brigitte von Rechenberg, Wiltrud Richter, Sebastien Hagmann

**Affiliations:** ^1^Center of Orthopaedic and Trauma Surgery/Spinal Cord Injury Center, Heidelberg University Hospital, Schlierbacher Landstraße 200a, Heidelberg 69118, Germany; ^2^Research Center for Experimental Orthopaedics, Heidelberg University Hospital, Schlierbacher Landstraße 200a, Heidelberg 69118, Germany; ^3^Leibniz Institute of Polymer Research Dresden, Hohe Straße 6, Dresden 01069, Germany; ^4^Chair of Functional Materials in Medicine and Dentistry and Bavarian Polymer Institute, University of Würzburg, Pleicherwall 2, Würzburg 97070, Germany; ^5^Musculoskeletal Research Unit (MSRU) Center for Applied Biotechnology and Molecular Medicine (CABMM), University Zürich, Winterthurerstraße 190, CH-8057 Zürich, Switzerland

## Abstract

Reinforced hydrogels represent a promising strategy for tissue engineering of articular cartilage. They can recreate mechanical and biological characteristics of native articular cartilage and promote cartilage regeneration in combination with mesenchymal stromal cells. One of the limitations of *in vivo* models for testing the outcome of tissue engineering approaches is implant fixation. The high mechanical stress within the knee joint, as well as the concave and convex cartilage surfaces, makes fixation of reinforced hydrogel challenging. *Methods*. Different fixation methods for full-thickness chondral defects in minipigs such as fibrin glue, BioGlue®, covering, and direct suturing of nonenforced and enforced constructs were compared. Because of insufficient fixation in chondral defects, superficial osteochondral defects in the femoral trochlea, as well as the femoral condyle, were examined using press-fit fixation. Two different hydrogels (starPEG and PAGE) were compared by 3D-micro-CT (*μ*CT) analysis as well as histological analysis. *Results*. Our results showed fixation of below 50% for all methods in chondral defects. A superficial osteochondral defect of 1 mm depth was necessary for long-term fixation of a polycaprolactone (PCL)-reinforced hydrogel construct. Press-fit fixation seems to be adapted for a reliable fixation of 95% without confounding effects of glue or suture material. Despite the good integration of our constructs, especially in the starPEG group, visible bone lysis was detected in micro-CT analysis. There was no significant difference between the two hydrogels (starPEG and PAGE) and empty control defects regarding regeneration tissue and cell integration. However, in the starPEG group, more cell-containing hydrogel fragments were found within the defect area. *Conclusion*. Press-fit fixation in a superficial osteochondral defect in the medial trochlear groove of adult minipigs is a promising fixation method for reinforced hydrogels. To avoid bone lysis, future approaches should focus on multilayered constructs recreating the zonal cartilage as well as the calcified cartilage and the subchondral bone plate.

## 1. Introduction

Osteoarthritis is known to be one of the fastest-growing causes of disability worldwide and significantly reduces the quality of life in affected individuals [1]. It is generally understood that once the disease has started, it may be delayed, but not cured. Due to an important number of cases where osteoarthritis starts from cartilage injuries or defects in young or middle-aged patients, the effective repair of these defects is a top-priority goal in orthopedic surgery.

To date, many approaches for cartilage repair have been proposed, including microfracture, osteochondral autograft transplantation, mosaicplasty, minced or micronized articular cartilage allografts, autologous matrix-induced chondrogenesis (AMIC), or autologous chondrocyte implantation (ACI) [2]. Nevertheless, none of these methods have been able to generate functional hyaline joint cartilage but rather induce fibrocartilage with inferior mechanical properties [2]. Another issue, especially for AMIC and ACI procedures, is the fixation of the constructs within the defect and to the adjacent native cartilage.

In more advanced osteoarthritis, the most commonly used treatment remains partial or total joint replacement. As a result of the limited durability of the implants and an ageing population, the revision rate is said to increase in the future. Also, it needs to be remembered that, despite the good functional and patient-reported outcome for certain joints such as the hip, other joints show significantly less promising results [3]. Also, implants remain a foreign body, with important issues such as the susceptibility for infections, and wear-related problems. For these reasons, cartilage repair remains an important task for orthopedic surgery and thus for research of cartilage repair.

Due to the low self-repair capacity of cartilage tissue, tissue engineering has focused on adequate cellular sources for cartilage repair. In contrast to articular chondrocytes (AC), mesenchymal stromal cells (MSCs) can be harvested with a smaller donor site morbidity at a higher expansion rate and therefore represent a promising cell source [4]. While the chondrogenic potential of MSCs has been demonstrated in numerous studies [5–7], their clinical use, however, is far from being widespread. One of the issues is that adequate carriers for the multitude of the MSCs effects in the cartilage defect and the joint seem to be a prerequisite for their use.

Hydrogels represent a promising approach as carriers for cell-based tissue engineering of cartilage tissue. They are easy to handle, can be evenly loaded with cells, and can promote chondrogenic differentiation of MSC [8, 9]. Hydrogels based on allyl-functionalized poly(glycidol) (P(AGE-co-G)) cross-linked thiofunctionalized hyaluronic acid (herein further named PAGE hydrogels) can be combined with mesenchymal stromal cells and showed promising results in *in vitro* chondrogenesis [9].

Besides, PEG-based hydrogels, such as starPEG, allow modulation of mechanical properties, degradability, and the customized incorporation of cysteine-containing peptides. They offer excellent conditions for MSCs regarding viability and proliferation. Furthermore, starPEG hydrogel constructs show good extracellular matrix distribution throughout the entire construct and promising results *in vivo* as well as *in vitro* [8, 10].

To address the mechanical and biological characteristics needed for cartilage regeneration, hydrogel reinforcement is necessary. Polycaprolactone (PCL) reinforcement can be used to achieve a similar stiffness as native articular cartilage [11]. PCL-reinforcement has also shown promising results for chondral and osteochondral regeneration *in vivo* [12, 13]. However, to date, *in vivo* studies with PCL-reinforced constructs in large animal models have been limited due to an insufficient fixation within the cartilage defect [13, 14].

Large animal models are an essential step to transfer successful *in vitro* tested strategies for cartilage repair to clinical applications. Many large animal models have been established in the past [2]. Minipigs show important similarities to the human species concerning knee anatomy as well as biomechanics. They are believed to predict clinical outcomes better than small laboratory animal models. Therefore, minipig models are a validated preclinical model for chondral and osteochondral repair [15]. Furthermore, minipigs are genetically similar individuals, which minimizes the interindividual inconsistencies in the healing potential [16]. These properties, as well as the similarity to the human knee joint concerning weight-bearing requirements and collagen fibre arrangement, make them a commonly used large animal model for cartilage regeneration [17–19]. Both trochlear groove and femoral condyle defect models have been established in minipigs, with important differences in biomechanical loading of the defect site [19, 20]. In clinical practice, both chondral and osteochondral defects come to attention. In contrast to chondral defects, osteochondral defects have the advantage of providing osseous integration and thus permanent ingrowth and fixation of the construct [18].

While, on the one hand, evaluation of safe handling of cell-laden constructs and their potential benefit in a clinical application should be generated from large animal studies, there remains, on the other hand, the question of safe fixation of the constructs. Huge variations in stability are observed depending on the fixation method used. Because of their unique properties, determination of proper fixation techniques cannot be performed in a general manner but must be considered for each construct combination individually. Obviously, one of the most important prerequisites for clinical use is that the constructs stay permanently in place, even when there is joint movement.

Therefore, the objective of this study was to adapt construct and fixation designs to obtain permanent retention of cell-laden tissue engineering constructs in a minipig animal model with cartilage defects. With fibrin glue being one of the most commonly used methods for fixation besides suture, or combinations of both, the main focus was to determine whether fibrin glue provides adequate fixation for different construct designs. However, other fixation techniques, such as press-fit fixation, were also evaluated. To determine whether trochlear groove or femoral condyle defects differed concerning fixation effectiveness, both chondral and osteochondral defects in both locations were compared. Lastly, because of the aforementioned important biomechanical differences in chondral and osteochondral defects, the effectiveness of fixation was evaluated upon defect depth, to define whether construct fixation in chondral defects can be as reliable as in osteochondral defects.

## 2. Materials and Methods

### 2.1. Animals

The experiments were performed on a total of 27 skeletally mature miniature pigs (Göttingen Minipigs, Ellegaard Göttingen Minipigs, Dalmose, Denmark; Mini-Lewe, Farm for Education and Research in Ruthe, University of Veterinary Medicine Hannover, Germany). 7 male and 20 female minipigs at an average age of 27.5 ± 4.8 months and with an average weight of 54.9 ± 10.4 kg at the day of surgery were used. Animals were kept in indoor runs allowing free movement and unrestricted access to water. They were fed once a day. The animal experiments were approved by the Animal Experimentation Committee Karlsruhe (G-203/14, G-117/16) and were performed according to the national guidelines for animal care in accordance with European Union Directive (2010/63/EU).

### 2.2. Isolation and Culture of Porcine Articular Chondrocytes

Porcine articular chondrocytes (pACs) were isolated from healthy porcine knee joints (n = 2 donors) from slaughter pigs obtained from the local abattoir as described before [21]. Briefly, rinsed cartilage was cut into small pieces, digested overnight with collagenase B (1.5 mg/ml, Roche Diagnostics, Basel, Switzerland) and hyaluronidase (0.1 mg/ml, Sigma-Aldrich, St. Louis, Missouri, USA), and filtered through a 40 *μ*m nylon mesh. Chondrocytes were seeded at 6000 cells/cm^2^ and expanded for 5-6 days in Dulbecco's Modified Eagle's Medium (DMEM) low glucose with L-glutamine, 10% fetal calf serum (FCS, Biochrom, Berlin, Germany), 100 U/mL penicillin, 100 µg/mL streptomycin at 37°C, and 6% CO_2_. The medium was replaced twice a week.

### 2.3. Isolation and Culture of Porcine Mesenchymal Stromal Cells

Porcine mesenchymal stromal cells (pMSCs) were isolated from bone marrow obtained by bone marrow aspiration of the pelvis from 2 minipig donors as described previously [22, 23]. The mononuclear cell fraction was separated by density gradient centrifugation using Ficoll®-Paque PLUS (GE Healthcare, Little Chalfont, United Kingdom), washed and seeded in an expansion medium (DMEM high glucose with L-glutamine, 10% FCS, 100 U/mL penicillin, and 100 *μ*g/mL streptomycin, supplemented with 4 ng/mL fibroblast growth factor-2 (FGF-2, Active Bioscience, Hamburg, Germany)). Mononuclear cells were plated at a density of 1.5 × 10^5^ cells/cm^2^ in monolayer culture, and adherent MSCs were subcultured for 3 passages (5 × 10^4^ cells/cm^2^) at 37°C and 6% CO_2_.

### 2.4. Preparation of Hydrogel Materials and Tissue Engineering Constructs

The starPEG hydrogel was formed by mixing thiol-end-functionalized (non-MMP-sensitive) starPEG or starPEG-MMP-conjugates carrying MMP-sensitive peptides at every arm and maleimide functionalized heparin of a constant molar ratio of (total) starPEG to heparin of 1.5 as described before [8, 24]. Hydrogel precursors were reconstituted with PBS. The total solid content of the hydrogel was adjusted to be constant at 5.3% after mixing of both components. The synthesis of linear P(AGE-co-G) and HA-SH was carried out as described before [9]. For hydrogel preparation, P(AGE-co-G) and HA-SH were dissolved in PBS with a final polymer concentration of 10 wt.% (5 wt.% of each polymer, and an equimolar ratio of SH and allyl groups) and the photoinitiator I2959 (BASF, Ludwigshafen, Germany; concentration of 0.05 wt.%) was added to the hydrogel precursor solution. The pH of the hydrogel precursor solution was neutralized using 5 M NaOH.

PCL (Purac Purasorb PC12, Corbion-Purac, Gorinchem, the Netherlands) was extruded using a RegenHU bioprinter (3D Discovery Gen1, RegenHU) equipped with a thermopolymer extruder (precision extrusion deposition print-head, screw-based extruder, HM110EX). A needle with an inner diameter of 0.25 mm was used (Nordson EFD). The reservoir of the extruder was heated up to 85°C, and the temperature at the print-head was set to 93°C. The material was preheated for 30 min before printing. PCL was printed with an extrusion speed of 17.5 revs/min, 3 bar extrusion pressure, and a collector plate speed of 5 mm/s. Crosshatched patterns of PCL strands (90°) were printed with a 6 × 6 mm circular footprint. The layer height was set to 0.20 mm, and the strand center to strand center distance was set to 1 mm. For the chondral approach, a 3-layered PCL was printed and 5-layered PCL was used in superficial osteochondral defects.

The hydrogel material was either used cell-free or pMSC, and pACs were added before polymerization. Hydrogels were polymerized in a disc mold without enforcement or enclosing either a CellCoTec (CCT; CellCoTec BV, Bilthoven, the Netherlands) or a polycaprolactone (PCL) enforcement (Supplemental Figure 1). The P(AGE-co-G) + HA-SH hydrogel solution was cross-linked using UV light (UVL hand lamp with filter, A. Hartenstein, Wuerzburg, Germany) at 365 nm for 5 min (1 mW cm^−2^). The PCL mesh had a diameter of 6 mm and a height of ≤0.6 mm with three layers for the chondral approach and a height of ≤1 mm with five layers for the osteochondral approach with a strand width of approximately 285 *μ*m. CCT and PCL were punched out with a diameter of 6 mm and a height of 0.6 mm (chondral approach) or 1 mm (superficial osteochondral approach). For enforced constructs, the respective enforcement was soaked with 25 *μ*L hydrogel in a disc mold. For nonenforced constructs, disc molds were filled with 30 *μ*L of hydrogel material. Constructs were created directly before implantation and, after polymerization, implanted using different fixation methods in chondral or osteochondral defects at the medial trochlear groove or the femoral condyles.

### 2.5. Study Design

Several fixation methods, based on clinically applied approaches, were tested for our precasted/nonreinforced as well as for CCT- and PCL-reinforced constructs until we achieved a reliable construct fixation.

First, the current gold standard fibrin glue (TISSEL, Baxter Deutschland GmbH, Unterschleißheim, Germany) was compared to BioGlue (CryoLife Inc., USA oder JOTEC aus Hechingen), which showed a superior implant fixation *in vitro* (data not shown). However, both fixation methods were insufficient for reliable construct retention.

Therefore, we tested direct suturing of the constructs as well as suturing of a collagen cover as described below. Despite a stable intraoperative fixation, we macroscopically and/or microscopically observed a postoperative implant dislocation after 4 weeks.

To evaluate whether different biomechanical properties have an influence on implant dislocation, implant fixation in the femoral trochlea was compared to the femoral condyle.

As none of these methods achieved reliable fixation in full-size chondral defects in either location, we decided to create a superficial osteochondral defect as described below. The additional height and stability of a superficial osteochondral defect allowed a reliable fixation of our construct using a press-fit technique. As there was no benefit of the femoral condyle as an alternative defect location, the further experiments were only evaluated for trochlear defects.

For study design and animal protection reasons, the sample sizes for ineffective fixation methods were kept to a minimum.

**Figure 1:**
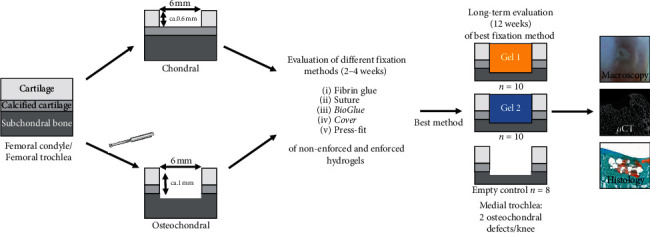
Study design.

After establishing a superficial osteochondral defect using press-fit fixation as a reliable fixation method, 2 different PCL-reinforced hydrogels, P(AGE-co-G) and starPEG hydrogels, were compared in terms of construct, retention, subchondral bone lysis, and cartilage formation (Figure 1) (Table 1).

Several fixation methods were tested for full-thickness chondral as well as superficial osteochondral defects in the femoral trochlea and femoral condyle of a minipig. After successful fixation of the implanted constructs, two different reinforced hydrogels (Gel 1: starPEG; Gel 2: PAGE) were compared using macroscopy scores, *μ*CT, and histological analysis.

### 2.6. Orthotopic Implantation

All operations were performed under aseptic conditions in endotracheal anesthesia (azaperone 5 mg/kg i.m., ketamine 15 mg/kg i.m., midazolam 0.5 mg/kg, propofol 2% i.v. to effect, and inhalation anesthesia with 1–3% isoflurane in oxygen). For orthotopic implantation at the medial facet of the trochlear groove of each femur, bilateral arthrotomy was performed as described before [25]. A straight median skin incision of 5–8 cm was set between the patellar apex and tibial tuberosity. After preparation of the subcutaneous tissue, the patella tendon was split longitudinally, giving access to the patellar space.

For implantation at the femoral condyles, arthrotomy was performed on the right hind leg with a 3 cm incision medially and laterally to the patella tendon, giving access to both femoral condyles.

Two defects with a diameter of 6 mm and a depth of about 0.6 mm (chondral approach) and 1 mm (osteochondral approach) were created either in the middle third of the medial facet of the trochlear groove of each femur or at the medial and lateral condyle of the right femur.

After marking the diameter with a biopsy punch, hyaline cartilage was detached with a curette until a full-thickness chondral defect with a smooth surface was achieved for implantation (chondral approach). For the osteochondral approach, after detachment of hyaline cartilage with the curette, the calcified layer was removed using a rose head drill at 40.000 rpm under constant irrigation without causing any bleeding of the subchondral bone. Four defects were created in each minipig and treated with different nonenforced or enforced hydrogel constructs or left empty as a control. Constructs were carefully inserted until their surface was just under the cartilage level and held in place by diverse fixation methods. Premilene 7/0 (B. Braun Melsungen AG, Melsungen, Germany) suture material was used for direct suturing of the constructs as well as fixation of a collagen cover (Chondro-Gide®, Geistlich Pharma AG, Wolhusen, Switzerland). The wound was closed by multilayered wound closure.

Postoperatively, the animals received buprenorphine every 12 hours (Buprenovet®, Bayer Vital GmbH, Leverkusen, Germany, 0.025 mg/kg i.m.) for a total of 48 hours as well as meloxicam (Metacam® 15 mg/ml, Boehringer Ingelheim Vetmedica GmbH, Ingelheim, Germany, 0.4 mg/kg p.o.) and amoxicillin (Duphamox® LA, Zoetis Deutschland GmbH, Berlin, Germany, 15 mg/kg i.m.) as long as required.

Minipigs were euthanized at 2, 4, or 12 weeks postoperatively under general anesthesia by an overdose of pentobarbital (Release®, WDT, Garbsen, Germany). Soft tissue was removed, and the joint capsule was opened to display the defect area. The defect area as well as the surrounding soft tissue was then examined for remaining constructs and cartilage or soft tissue irregularities in shape and colour. After macroscopic examination and photo documentation, the trochlea or condyles were detached and defect areas were processed for analyses.

### 2.7. Micro-CT Analysis

Micro-CT analysis of the defect site was performed directly after explantation using a SkyScan 1076 *in vivo* X-ray microtomography (SkyScan, Bruker-micro-CT, Belgium). The following settings were used: 1.0 mm aluminum filter, source voltage 100 kV, source current 100 *μ*A, exposure time 400 ms, voxel size 18 *μ*m, and rotation step 0.5 degree. Reconstruction of images was performed using NRecon® software (version 1.6.3.2, SkyScan). In case of bone analyses, CTAn® (version 1.13.2.1, SkyScan) was used for calculation of the volume of bone tissue within a defined volume of interest (VOI, 8 mm × 1 mm) represented by the defect borders and 58 sectional images into the bone. The lower grey level was set at 80, and the upper grey level was set at 255. To measure the effects on bone volume/total volume in each group, one animal with four empty defects was explanted directly after surgery. Micro-CT analysis was performed to generate representative day 0 values to compare with the outcome in each group after 12 weeks.

### 2.8. Histology

Directly after micro-CT imaging, porcine osteochondral samples were fixed for 72 h in 4% formaldehyde followed by either decalcification for up to 16 weeks in ethylenediaminetetraacetic acid or 13–15 days in Formical® 2000 (StatLab, USA) (*n* = 30), respectively, or processed for polymethylmethacrylate (PMMA) embedding (*n* = 46). Decalcified specimens were dehydrated and dissolved in acetone and xylene for PCL remnants, before samples were embedded in paraffin. After cutting 5 *μ*m serial sections, type II collagens were stained with mouse anti-human monoclonal antibodies (MP Biomedicals, Germany; Jackson ImmunoResearch, UK) according to standard immune histology protocols with ImmPACT Vector Red counterstaining (Vector Laboratories, USA).

PMMA embedded specimens were stained using toluidine blue. Polymerization was completed in an incubator at 37.5°C. Ground sections were cut, mounted on Acropal slides and polished before surface staining with toluidine blue.

### 2.9. Modified O'Driscoll Score

For semiquantitative histomorphological evaluation of repair quality of the 12-week groups, one representative toluidine blue stained section was assessed by two blinded observers according to criteria of a modified O'Driscoll score [26] (Supplemental Table 1).

### 2.10. Statistics

The Shapiro–Wilk test was performed to confirm normal distribution of all our data. Descriptive statistics were performed for all continuous variables with mean and standard deviation (SD). To investigate the difference between groups, one-way ANOVA was used and corrected with the Bonferroni test. Histological data of all groups were compared using a Kruskal–Wallis test with the post hoc Mann–Whitney U-test and signed-rank test. All tests were performed in accordance with two-sided testing. Differences were considered to be significant at *p* ≤ 0.05. Statistical evaluation was accomplished using SPSS (version 22.0.0, SPSS Inc., Chicago, IL, USA).

## 3. Results

All minipigs recovered well from anesthesia. After five to nine days of postoperative analgesia and antibiotic treatment, all animals were able to walk without any clinical signs of pain or lameness. The minipigs stayed healthy, and no unforeseen events were encountered until euthanasia up to 12 weeks after surgery.

### 3.1. Failed Fixation of Hydrogel Constructs in a Trochlea Groove Chondral Defect

To develop the best fixation method for a cell-free precasted hydrogel, several clinically or experimentally used approaches were compared in a chondral trochlea defect *in vivo*.

Precasted hydrogels (*n* = 4), as well as CCT-reinforced hydrogels (*n* = 8), were fixed using fibrin glue. After 4 weeks, none of the constructs were detectable within any of the defects.

**Figure 2:**
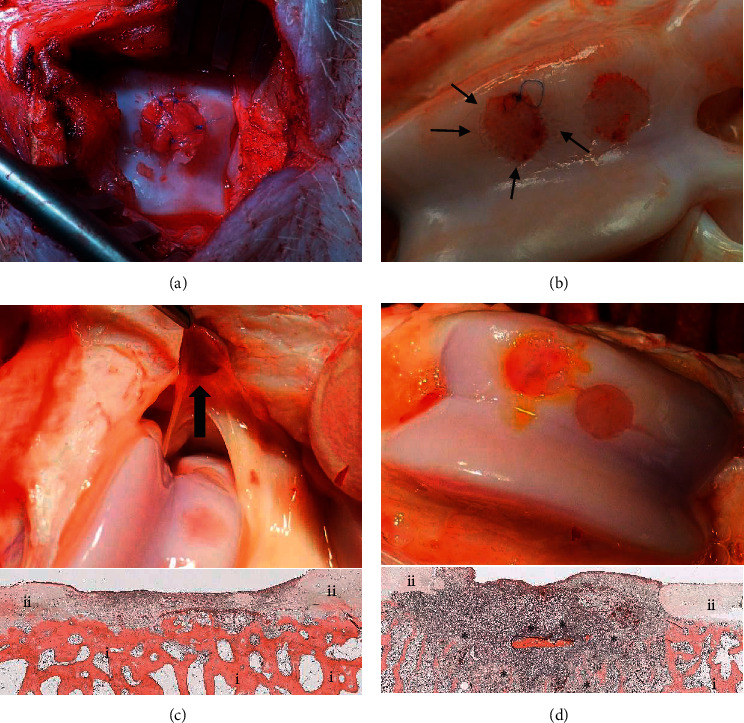
Fixation of novel constructs in chondral trochlea defects. Covering the construct with Chondro-Gide® resulted in intraoperative squeeze out of the hydrogel (a). CCT-reinforced hydrogels were directly sutured to the surrounding cartilage. None of the constructs remained in place after 4 weeks. Macroscopic changes to the surrounding cartilage due to suture tear out (thin arrow) were observed (b). Fibrin glue was used to fix a precasted hydrogel with CCT-reinforcement in a 6 mm trochlea defect. After 4 weeks, the displaced construct was found within the synovial tissue (big arrow), H and E staining (c). BioGlue® led to macroscopic colour changes of the surrounding cartilage as well as microscopic bone lysis (H&E staining) of the subchondral bone plate (*∗*) (d). Scale bar: 2 mm; *i*: bone; *ii*: cartilage; *∗*: bone lysis.

Chondro-Gide® was used as a covering flap to prevent displacement of a nonreinforced construct (*n* = 1). Intraoperatively, due to the fragility of the hydrogel, the pressure applied to the cover pushed the gel out of the defect (Figure 2(a)). While the Chondro-Gide® remained in place after 4 weeks, no remnants of the gel were found within the defect neither macroscopically nor microscopically.

The CCT-reinforcement was stable enough for direct suturing to the surrounding cartilage. While we obtained a stable fixation intraoperatively, none of the constructs thus treated remained in place after 4 weeks *in vivo* (*n* = 3) (Figure 2(b)).

However, in several knees, the dislocated construct was found within the surrounding tissue, suggesting an early dislocation rather than an early, full degradation of the construct within the first 4 weeks. There was no evidence for a macroscopic inflammatory cartilage reaction. Histologically, there were no subchondral lesions in any of the defects and no remaining hydrogel and reinforced hydrogel detectable (Figure 2(c)).

*In vitro* experiments had shown a superior adhesion of constructs glued with BioGlue® compared to fibrin glue (Supplemental Figure 2). Despite the promising *in vitro* results, none of the constructs fixed with BioGlue remained in place after 4 weeks (*n* = 4). At the time of explantation, a macroscopic change in colour of the surrounding cartilage was observed. These findings correlated with microscopic results that showed degeneration of the surrounding cartilage but also severe lysis of the cortical structure of the subchondral bone in all BioGlue® treated defects (Figure 2(d)). Micro-CT visualization revealed bone erosion after the use of BioGlue® in chondral defects (data not shown).

In conclusion, none of the tested methods was able to reproducibly fix a precasted (reinforced) hydrogel in a chondral defect of the trochlea groove.

### 3.2. No Influence of Defect Location on Retention of the Construct

Due to the constant shearing forces in the femoropatellar joint, the femoral condyle was tested as an alternative defect location with different biomechanical loading. The high stiffness and fragility of the CCT-reinforcement deemed inapplicable for our application. PCL-reinforcement was easier to handle and less fragile with a good porous system (Supplemental Figure 1). Furthermore, pACs were added to our constructs. After 4 weeks *in vivo*, histological analysis showed no hydrogel remnants within the defects treated without PCL-reinforcement in either location (*n* = 4 trochlea; *n* = 2 condyle). PCL-reinforcement resulted in a higher retention rate of 2/3 on the trochlea and 1/2 on the femoral condyles. Macroscopically, the trochlea defects were surrounded by fewer fissures and changes in the native cartilage compared to the condylar defects.

Subtle changes of the subchondral bone, without osteolysis, were observed in trochlear defects treated with PCL-reinforced constructs. Less remodeling of the subchondral bone was found in the defect with a dislocated PCL-reinforced construct, where a fibrous tissue layer formed within the defect. The PCL structure was still definable after 4 weeks.

**Figure 3:**
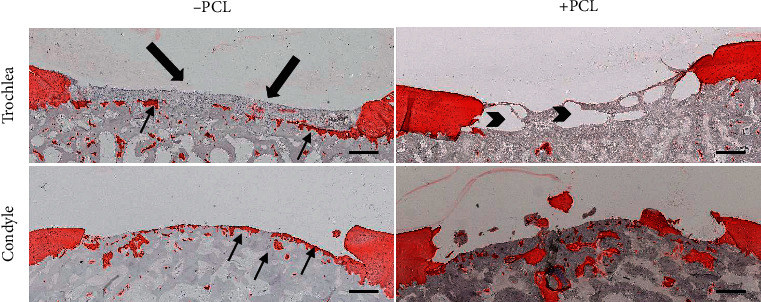
Insufficient construct retention in condylar and trochlea chondral defects with and without PCL-reinforcement. Collagen type II staining of exemplary depiction of one defect per group after 28 days. Both defects without PCL (left) showed a residual layer of cartilage (arrow). Fibrous tissue formed in one of the defects (big arrow). Fibrous tissue was retained in between PCL strands. Retained PCL-construct (arrowhead). Scale bar: 500 *μ*m.

A lower retention rate, as well as microscopically formation of less fibrous tissue within the defects treated without PCL-reinforcement, was observed in femoral condyle defects compared to trochlea defects, which might partially have been caused by the convex surface of the femoral condyle (Figure 3).

To conclusively define whether the trochlea grove offers a satisfactory retention of PCL-reinforced constructs in a chondral defect, further 12 defects were tested. However, after 2 weeks, all 12/12 defects showed no PCL and or hydrogel remnants within the defect.

Thus, a pure chondral defect did not provide the necessary defect depth for a reliable fixation of nonenforced and enforced constructs.

### 3.3. Improved Construct Fixation in a Superficial Osteochondral Defect

By creating a superficial osteochondral defect without significant bleeding points by drilling with a rose head drill, we were able to achieve a defect depth of approximately 1 mm. The stability of the subchondral bone plate as well as the extra height allowed a press-fit fixation of the pAC containing PCL-reinforced constructs in combination with fibrin glue. Histological analysis confirmed that 8/8 of PCL-reinforced constructs were still detectable after 4 weeks, while 0/8 of the nonreinforced constructs (one of the constructs had an additional Chondro-Gide® cover) was detectable.

**Figure 4:**
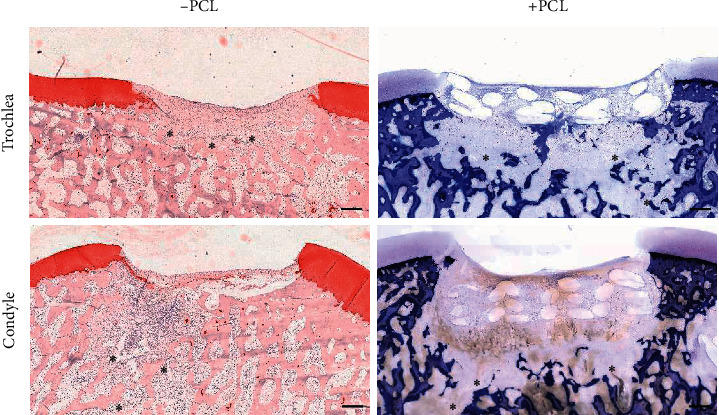
Histological evaluation of the defects 4 weeks after implantation into trochlea and condylar defects. Collagen type II staining of nonreinforced constructs. Toluidine blue staining of PMMA embedded PCL containing defects. In all osteochondral defects, regardless of PCL-reinforcement, osteolysis of the subchondral bone was found. *∗*: bone lysis. Scale bar: 500 *μ*m.

Due to difficulties in paraffin fixation, caused by the PCL-construct, explants were fixed in PMMA for histology. Histological analysis revealed osteolysis of the subchondral bone regardless of PCL-reinforcement (Figure 4).

**Figure 5:**
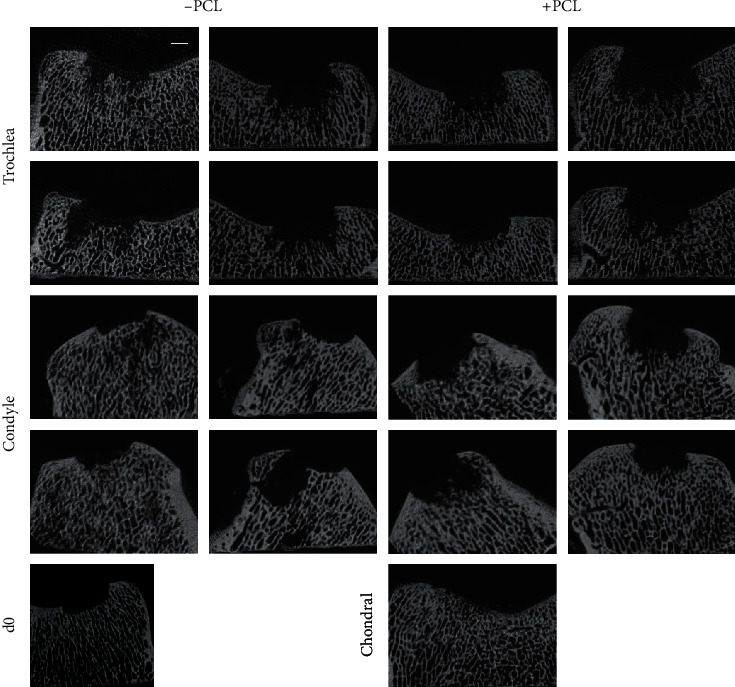
*μ*Ct visualization of bone lysis 4 weeks after implantation of nonreinforced and PCL-reinforced constructs in superficial osteochondral defects. Severe bone lysis was found in all defects regardless of the implanted construct. A reference d0 scan is shown, indicating the original defect depth, as well as a chondral defect showing no bone lysis. Scale bar: 2 mm.

To illustrate the extent of the bone lysis, we performed micro-CT scans. In both groups, independently of the location, severe osteolysis was found compared to day 0 scans. Osteolysis was found in both osteochondral defects, treated with PCL-reinforced and nonreinforced constructs. In contrast, chondral defects did not show any signs of osteolysis in micro-CT scans (Figure 5). Due to the lack of obvious benefit and the observed changes in the adjacent cartilage, the use of defects in the femoral condyles was aborted.

After establishing press-fit fixation as an applicable method for our precasted gels, two different hydrogels (starPEG and PAGE) were tested to determine whether the use of a different hydrogel had an effect on bone lysis. Furthermore, we proceeded without the additional use of fibrin glue due to potential immunological effects, which may have been a potential source for the observed changes in the subchondral bone. We therefore quantified bone lysis in both hydrogel groups as well as empty controls using micro-CT scans and compared the findings to day 0 defects.

### 3.4. Successful Press-Fit Fixation of PCL-Reinforced, pMSC Containing Constructs with Two Different Hydrogel Materials (StarPEG vs. PAGE vs. Empty Controls) in Osteochondral Defects of the Trochlear Groove

At explantation after 12 weeks, no signs of inflammation, intra-articular pathological changes, or adverse reactions were observed within the joints or the surrounding tissues. Macroscopically, PCL was clearly visible in 17/20 defects after 12 weeks *in vivo*. Five of the visible enforcements showed spiky ends of their top layer, indicating inceptive deterioration of PCL enforcement. All 3 defects with macroscopically indiscernible PCL belonged to the starPEG group. All empty defects were filled with white repair tissue.

### 3.5. Increased Bone Loss in Defects Treated with PCL-Reinforced Constructs Compared to Empty Control Defects in a 3D-Micro-CT Analysis

**Figure 6:**
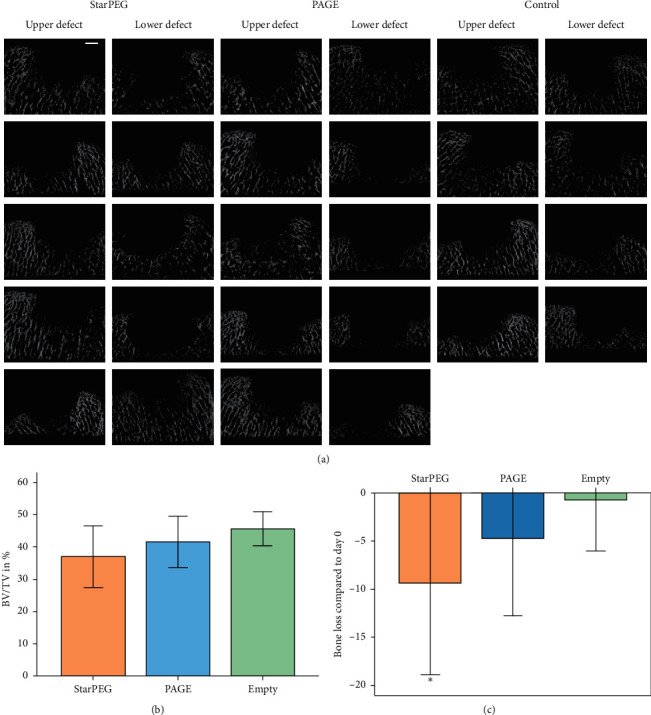
*μ*CT scans of osteochondral trochlea defects 12 weeks after implantation of different hydrogels. *μ*CT visualization revealing bone lysis in all groups, scale bar: 2 mm (a). Bone volume/total volume was quantified in a volume of interest for each defect, and there was no statistical significance between the groups (b). To depict the loss of bone volume, BV/TV was compared to day 0 confirming more severe bone loss in defects treated with PCL-reinforced constructs compared to empty control defects. Mean percentage of bone loss ± SD per group compared to day 0 defects. *∗*: *p* ≤ 0.05 vs. empty control (ANOVA) (c).

To assess the underlying bone changes associated with defect treatment, all defect sites underwent micro-CT imaging directly after explantation. Micro-CT visualization revealed bone erosion in all groups, which seemed more pronounced in groups treated with PCL enforcement compared to empty controls (Figure 6(a)).

Bone volume/total volume (BV/TV) was quantified in a volume of interest for each defect of the 12-week study under standardized conditions. The mean of BV/TV of the three groups was 37.05% (starPEG: 18.39–49.24%), 41.65% (PAGE: 30.45–57.74%), and 46.41% (empty controls: 35.84–52.70%) (Figure 6(b)). Statistical analysis (ANOVA, Bonferroni correction, *p* ≤ 0.05) revealed no significant differences between the groups. After 12 weeks, the mean BV/TV decreased by 9.36% in the starPEG, 4.76% in the PAGE group, and by 0.73% in the empty control group compared to 4 empty defects explanted directly after surgery (day 0) (Figure 6(c)). Therefore, treatment with PCL-reinforced constructs with PAGE tended to induce more bone loss than empty controls while bone loss was significantly higher in the starPEG group compared to empty controls (ANOVA, LSD, *p*=0.031) (Figure 6(c)).

### 3.6. More Cell-Containing Defects in the StarPEG Group Compared to the PAGE Group

Sections of polymethylmethacrylate (PMMA) embedded samples were stained for proteoglycans. Microscopically, PCL-reinforcement remained in 19/20 defects, indicating the loss of only one construct at 12 weeks. This defect showed severe bone lysis and belonged to the starPEG group. Overall, the integration strategy was therefore successful.

Hydrogel fragments were found in 6/10 defects in the starPEG-treated group and 6/10 defects in the PAGE-treated group. In the starPEG group, 5/6 defects with hydrogel fragments were cell-containing compared to only 3/6 in the PAGE group.

### 3.7. Heterogenous Regeneration Tissue in the Histological Evaluation of all Groups

Regeneration was graded by two blinded observers using a modified O´Driscoll score covering the nature of the predominant tissue (cell morphology and staining of matrix) and structural characteristics (surface, structure, lateral and basal integration, and subchondral bone) (Supplemental Table 1).

**Figure 7:**
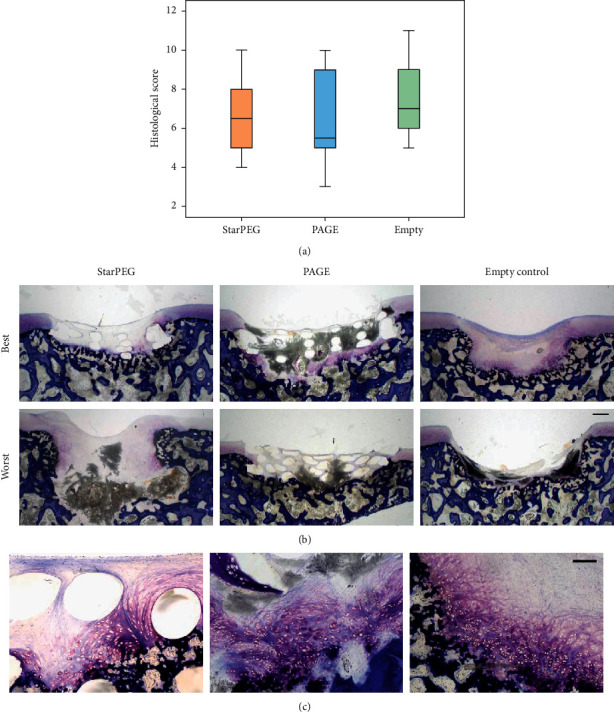
Histology after 12 weeks *in vivo*. Histological evaluation using modified O‘Driscoll score by two blinded observers. Boxes represent first and third quartiles, medians are given as horizontal lines, and whiskers are maximal and minimal values. StarPEG and PAGE *n* = 10, empty *n* = 4, and there was no statistical significance between the groups, *∗*: *p* ≤ 0.05 vs. empty control (ANOVA) (a). Best and worst results per group according to histological scoring (mod. O‘Driscoll score done by two blinded observers). Toluidine blue staining; scale bar: 500 *μ*m (b). Magnification of best constructs per group regarding cell morphology and metachromasia, toluidine blue staining; scale bar: 200 *μ*m (c).

Overall, cartilage regeneration was very heterogeneous with score ranges of 5–11 (empty controls), 4–10 (starPEG), and 3–10 (PAGE). Due to this heterogeneity, no statistical significant difference was obtained between the groups (Figure 7(a)).

The defects were mostly filled with fibrous proteoglycan-negative regeneration tissue (Figure 7(b)). Several defects clearly showed good cartilage regeneration developing from the underlying bone (Figure 7(c)).

## 4. Discussion

Most studies addressing chondral regeneration use osteochondral defect models to achieve a reliable construct ingrowth and integration. The defect depth of osteochondral defects in minipig models usually ranges from 5 mm to 10 mm [15, 17, 27, 28]. The osseous trauma might be associated with the formation of bone cysts due to an inflow of synovial fluid or accelerated bone resorption, even up to 12 months after implantation [20, 29, 30]. Femoral condyle defects show higher cartilage repair, but also more subchondral bone cyst formation and increased postoperative pain [20]. Only a few studies have focused on regeneration of a full-size chondral defect without creating an additional osseous trauma. This may be due to the fact that the thin cartilage of <1 mm in most large animal models makes retention of a precasted construct difficult [31]. Despite similar mechanical properties, cartilage thickness differs from 1.5 mm in the porcine knee compared to 2.35 mm in humans [16, 32]. Previous studies have shown even thinner cartilage layers in minipigs of approximately 0.5 mm [17].

There is general consensus that creates layered constructs that mimic the native cartilage is crucial for cartilage repair. To allow successful treatment of cartilage defects, however, retaining these constructs is essential. To address this issue, we tested several fixation methods *in vivo*.

Fibrin glue is commonly used in experimental as well as in clinical settings for ACI. Previous studies mainly focused on direct injection of a cell-loaded fibrin- or hydrogel construct into osteochondral or chondral defects, with some of the constructs covered with a collagen membrane [25, 33]. Fixation of constructs in chondral defects is far more difficult compared to osteochondral defects. In an animal model, even after 4 week immobilisation, neither fibrin glue nor fibrin glue combined with a periosteal flap achieved sufficient implant fixation in a chondral defect.

As shown in our experiments, fibrin glue alone was not sufficient to retain a precasted construct within a trochlear or condylar chondral defect.

BioGlue® is a surgical glue that is already in clinical use, especially in the field of cardiac and vascular surgery. It aims to achieve hemostasis and to seal and reinforce soft tissue or damaged parenchyma. Despite its clinical application, *in vitro* studies have shown cytotoxic and proinflammatory effects, such as macrophage activation [34]. Macroscopic as well as microscopic findings in our study reveal an obvious degeneration of the surrounding cartilage as well as severe lysis of the cortical structure of the subchondral bone. We thus conclude that BioGlue® is not suitable for fixation in cartilage repair, most likely due to the effects of the glutaraldehyde component [35].

To determine whether the fixation failure was mainly caused by the biomechanical loading of the trochlear groove, we compared osteochondral defects positioned in the trochlear groove and the femoral condyles. There was no significant difference in fixation stability between the trochlear groove and the medial femoral condyle. Osteochondral defects in the trochlear groove are known to lead to less subchondral cyst formation and are therefore the preferred defect location compared to condyle defects. However, they also seem to bear the disadvantage of an inferior capacity to form cartilage-like tissue. In cartilage regeneration studies, the lower self-repair capacity is of advantage to emphasize the effect of the implanted construct compared to a control defect. These differences in healing capacity are most likely due to the different biomechanical properties of both locations [20, 36]. The flatter surface of the trochlear groove compared to the anatomy of the femoral condyles is another advantage for the experimental setting.

To avoid the additional trauma of a deep full-size osteochondral defect, we created a defect of approximately 1 mm depth concurrent with our construct height. Therefore, we removed the chondral layer, including the calcified cartilage as well as approximately 0.5 mm of the subchondral bone plate, creating a superficial osteochondral defect.

Creating a superficial osteochondral defect enabled us to obtain the necessary depth for a press-fit fixation of our construct, with only minor bleeding points. Another study that created superficial osteochondral defects used a periosteal flap, fibrin glue, and/or platelet-rich plasma to hold the engineered cartilage construct in place. In this study, the periosteal flap received the best results after 2 months. However, most implants that were glued in place without a cover were lost despite an initial 4-week phase of restricted joint movement [37], which is consistent with our findings. In our study, due to the stiffness of the reinforced construct or the fragility of the hydrogel, covering did not increase the retainment of the construct. A study by Mainil-Varlet created superficial osteochondral defects and was able to reduce implant loosening of press-fit fixed constructs compared to chondral defects. Similar to our result, they observed a high loosening rate in chondral defects of ∼50%, compared to only 20% in superficial osteochondral defects [38]. In our study, the loosening rate was even lower, with only 5% of the constructs loosened after 12 weeks.

Previous studies have shown that the use of fibrin glue led to distinctive cell infiltration in the subchondral bone and inferior tissue quality compared to a fibrin glue-free control group [37]. Absence of an adhesive like fibrin glue allowed us to avoid any biocompatibility issues except for those from the construct itself. This may be especially important due to the fact that fibrin glue has shown to produce negative effects such as inferior tissue quality, lower defect filling, and fibrocartilage formation [14, 37].

PCL-collagen combined with BMSC has shown to have a positive effect on morphological outcomes of cartilage-like repair tissue [36]. Furthermore, PCL has been used for subchondral bone regeneration in combination with tricalcium phosphate (TCP) [39]. In our opinion, the PCL-reinforcement is necessary to provide higher stiffness, thus allowing a press-fit fixation into the superficial osteochondral defect. In addition, giving the construct more flexibility and stability compared to CCT, PCL should also provide an adequately porous system, allowing better integration of the scaffold. In our study, this allowed a higher retention rate in chondral defects compared to CCT-reinforcement. Studies have shown that stiff constructs improve bone regeneration more than soft scaffolds [40]. In contrast to our study, Mancini et al. did not achieve a reproducible retention with PCL-reinforced hydrogels in horses, but only with PCL anchors. The authors created a deep osteochondral defect for press-fit fixation of a PCL-reinforced construct into the defect. They observed massive inflammation within the first 4 weeks after surgery, which most likely caused severe bone loss, irrespective of the hydrogel used. As an explanation, the authors discuss that these effects may have been caused by a xenogenic glue. This was an effect that we were able to eliminate in our final study. The bone loss observed in our study, therefore, may have been attributed to mechanical stress, which might have been enhanced due to the stiffness of our PCL-reinforcement. While the defects were drilled under constant irrigation and there was no indication for intraoperative heat generation, we cannot reliably exclude an effect of the drilling process on the observed bone lysis.

The relatively short observation period of <3 months may have been insufficient to show full remodeling of the subchondral bone [14]. However, previous studies with osteochondral defects have also shown the presence of bone cysts after one year [20]. Techniques that expose the subchondral bone, such as the implantation of osteochondral scaffolds or osteochondral allografts, may induce subchondral bone cyst formation either through fluid intrusion or bony contusion, as has been shown in an ovine defect model [41]. Nevertheless, bone remodeling and or loss of bone matrix has also been described for chondral defects [42].

Friedman et al. tried to identify a reliable fixation for a woven PCL scaffold in a chondral defect using press-fit, fibrin glue, trans-chondral suture fixation, or subchondral anchor fixation, in combination with microfracture of the subchondral bone. They were able to achieve reliable retention of the scaffold with subchondral anchoring, while none of the other methods showed reproducible results (<50%). The insufficient retention in the press-fit group was mainly attributed to the woven, flexible structure of the PCL-based construct. In combination with the PCL, the authors observed subchondral bone lysis surrounding the anchor [13].

In our study, there was no significant difference in cartilage repair between the two hydrogels (starPEG and PAGE) and the empty control defects, indicating no significant benefit of defect treatment after 12 weeks. However, in the starPEG group, more hydrogel fragments were found within the defect area, and more of these hydrogel fragments were cell-containing. These fragments may thus have a longer effect and impact on the regeneration process. Bone loss, on the other hand, was similar in both hydrogels. StarPEG, therefore, seems to be a more promising hydrogel for future studies [43].

The use of nonzonal constructs did not lead to effective cartilage restoration in our study. Restoration of the subchondral bone plate, as well as the calcified cartilage, however, is deemed essential for a functional cartilage healing.

In order to pave the way for cartilage repair from bench to bedside, the constructs must prove functional in daily clinical routine and imperatively have to be easy to implant and show reliable fixation. Press-fit fixation meets these requirements and has been clinically tested for osteochondral defects [44]. As a result of the insufficient cartilage thickness in minipigs, a superficial osteochondral defect was essential for press-fit fixation of our constructs. The greater cartilage thickness of human cartilage might be sufficient for press-fit fixation without creating an additional osteochondral defect. However, removal of the calcified cartilage might prove important in osteoarthritis due to the fact that the subchondral plate is usually thickened in osteoarthritis, causing a different mechanical property [45]. In these cases, removing the subchondral plate and restoring its properties might be essential for successful treatment of OA, making the proposed superficial osteochondral defect model a promising large animal model for future studies. Albeit, more studies are needed before introducing actual strategies combining constructs, cells, growth factors, and fixation into clinical use.

## 5. Conclusions

Superficial osteochondral defects allowed successful press-fit fixation of PCL-reinforced hydrogel constructs in the medial trochlear groove of adult minipigs. In chondral defects, no technique provided adequate fixation in this model. Defects treated with these constructs showed enhanced osteolysis in the micro-CT analysis. Nonzonal constructs did not prove superior to empty control defects.

## Figures and Tables

**Table 1:** Overview of the number of defects per group and fixation method for the 2–4 week study. 12 minipigs with trochlea chondral defects with reinforced hydrogels and fibrin glue fixation were euthanized after 2 weeks, and all other animals were euthanized after 4 weeks.


	Trochlea				Condyle			
	Chondral		Osteochondral		Chondral		Osteochondral	
Reinforcement	−	+	−	+	−	+	−	+
Fibrin glue	4	23	4		2	2	3	
BioGlue®		4						
Fibrin + cover	1						1	
Direct suture		3						
Press-fit + fibrin glue				4				4

## Data Availability

The histological and *μ*CT data used to support the findings of this study are available from the corresponding author upon request.

## Supplementary Materials

Supplementary Materials Supplemental Figure 1: characterisation of the used PCL enforcement. Supplemental Figure 2: *in vitro* pullout strength comparison of fibrin glue and BioGlue® for CCT-reinforced PAGE hydrogels. Supplemental Table 1: modified O'Driscoll score as used for semiquantitative histomorphological evaluation.

## References

[1] Conaghan P. G., Porcheret M., Kingsbury S. R. (2015). Impact and therapy of osteoarthritis: the arthritis care OA nation 2012 survey. *Clinical Rheumatology*.

[2] Anderson J. A., Little D., Toth A. P. (2014). Stem cell therapies for knee cartilage repair. *The American Journal of Sports Medicine*.

[3] O’Brien S., Bennett D., Doran E., Beverland D. E. (2009). Comparison of hip and knee arthroplasty outcomes at early and intermediate follow-up. *Orthopedics*.

[4] Wagner W., Wein F., Seckinger A. (2005). Comparative characteristics of mesenchymal stem cells from human bone marrow, adipose tissue, and umbilical cord blood. *Experimental Hematology*.

[5] Goepfert C., Slobodianski A., Schilling A. F., Adamietz P., Pörtner R. (2010). Cartilage engineering from mesenchymal stem cells. *Bioreactor Systems for Tissue Engineering II*.

[6] Griffin M., Hindocha S., Khan W. S. (2012). Chondrogenic differentiation of adult MSCs. *Current Stem Cell Research and Therapy*.

[7] Le H., Xu W., Zhuang X., Chang F., Wang Y., Ding J. (2020). Mesenchymal stem cells for cartilage regeneration. *Journal of Tissue Engineering*.

[8] Hesse E., Freudenberg U., Niemietz T. (2018). Peptide-functionalized starPEG/heparin hydrogels direct mitogenicity, cell morphology and cartilage matrix distribution in vitro and in vivo. *Journal of Tissue Engineering and Regenerative Medicine*.

[9] Stichler S., Bock T., Paxton N. (2017). Double printing of hyaluronic acid/poly(glycidol) hybrid hydrogels with poly(epsilon-caprolactone) for MSC chondrogenesis. *Biofabrication*.

[10] Kunisch E., Knauf A. K, Hesse E (2018). StarPEG/heparin-hydrogel based in vivo engineering of stable bizonal cartilage with a calcified bottom layer. *Biofabrication*.

[11] Visser J., Melchels F. P. W., Jeon J. E. (2015). Reinforcement of hydrogels using three-dimensionally printed microfibres. *Nature Communications*.

[12] Christensen B. B., Foldager C. B., Hansen O. M. (2012). A novel nano-structured porous polycaprolactone scaffold improves hyaline cartilage repair in a rabbit model compared to a collagen type I/III scaffold: in vitro and in vivo studies. *Knee Surgery, Sports Traumatology, Arthroscopy*.

[13] Friedman J. M., Sennett M. L., Bonadio M. B. (2018). Comparison of fixation techniques of 3D-woven poly(*ϵ*-caprolactone) scaffolds for cartilage repair in a weightbearing porcine large animal model. *Cartilage*.

[14] Mancini I. A. D., Vindas Bolaños R. A., Brommer H. (2017). Fixation of hydrogel constructs for cartilage repair in the equine model: a challenging issue. *Tissue Engineering Part C: Methods*.

[15] Christensen B. B., Foldager C. B., Olesen M. L. (2015). Experimental articular cartilage repair in the Göttingen minipig: the influence of multiple defects per knee. *Journal of Experimental Orthopaedics*.

[16] Chu C. R., Szczodry M., Bruno S. (2010). Animal models for cartilage regeneration and repair. *Tissue Engineering Part B: Reviews*.

[17] Gotterbarm T., Breusch S. J., Schneider U., Jung M. (2008). The minipig model for experimental chondral and osteochondral defect repair in tissue engineering: retrospective analysis of 180 defects. *Laboratory Animals*.

[18] Gotterbarm T., Richter W., Jung M. (2006). An in vivo study of a growth-factor enhanced, cell free, two-layered collagen-tricalcium phosphate in deep osteochondral defects. *Biomaterials*.

[19] Jung M., Tuischer J. S., Sergi C. (2006). Local application of a collagen type I/hyaluronate matrix and growth and differentiation factor 5 influences the closure of osteochondral defects in a minipig model by enchondral ossification. *Growth Factors*.

[20] Jung M., Breusch S., Daecke W., Gotterbarm T. (2009). The effect of defect localization on spontaneous repair of osteochondral defects in a Göttingen minipig model: a retrospective analysis of the medial patellar groove versus the medial femoral condyle. *Laboratory Animals*.

[21] Benz K., Breit S., Lukoschek M., Mau H., Richter W. (2002). Molecular analysis of expansion, differentiation, and growth factor treatment of human chondrocytes identifies differentiation markers and growth-related genes. *Biochemical and Biophysical Research Communications*.

[22] Bertram H., Boeuf S., Wachters J. (2009). Matrix metalloprotease inhibitors suppress initiation and progression of chondrogenic differentiation of mesenchymal stromal cells in vitro. *Stem Cells and Development*.

[23] Haynesworth S. E., Barer M. A., Caplan A. I. (1992). Cell surface antigens on human marrow-derived mesenchymal cells are detected by monoclonal antibodies. *Bone*.

[24] Tsurkan M. V., Chwalek K., Prokoph S. (2013). Defined polymer-peptide conjugates to form cell-instructive starPEG-heparin matrices in situ. *Advanced Materials*.

[25] Jung M., Kaszap B., Redöhl A. (2009). Enhanced early tissue regeneration after matrix-assisted autologous mesenchymal stem cell transplantation in full thickness chondral defects in a minipig model. *Cell Transplantation*.

[26] O’Driscoll S. W., Keeley F. W., Salter R. B. (1988). Durability of regenerated articular cartilage produced by free autogenous periosteal grafts in major full-thickness defects in joint surfaces under the influence of continuous passive motion. A follow-up report at one year. *The Journal of Bone and Joint Surgery. American Volume*.

[27] Sheu S. Y., Wang C. H., Pao Y. H. (2017). The effect of platelet-rich fibrin on autologous osteochondral transplantation: an in vivo porcine model. *The Knee*.

[28] Hsieh Y.-H., Shen B.-Y., Wang Y.-H., Lin B., Lee H.-M., Hsieh M.-F. (2018). Healing of osteochondral defects implanted with biomimetic scaffolds of poly(*ε*-caprolactone)/hydroxyapatite and glycidyl-methacrylate-modified hyaluronic acid in a minipig. *International Journal of Molecular Sciences*.

[29] von Rechenberg B., Akens M. K., Nadler D. (2003). Changes in subchondral bone in cartilage resurfacing-an experimental study in sheep using different types of osteochondral grafts. *Osteoarthritis and Cartilage*.

[30] Orth P., Goebel L., Wolfram U. (2012). Effect of subchondral drilling on the microarchitecture of subchondral bone. *The American Journal of Sports Medicine*.

[31] Ahern B. J., Parvizi J., Boston R., Schaer T. P. (2009). Preclinical animal models in single site cartilage defect testing: a systematic review. *Osteoarthritis and Cartilage*.

[32] Klein T. J., Malda J., Sah R. L., Hutmacher D. W. (2009). Tissue engineering of articular cartilage with biomimetic zones. *Tissue Engineering Part B: Reviews*.

[33] Niemietz T., Zass G., Hagmann S., Diederichs S., Gotterbarm T., Richter W. (2014). Xenogeneic transplantation of articular chondrocytes into full-thickness articular cartilage defects in minipigs: fate of cells and the role of macrophages. *Cell and Tissue Research*.

[34] Umashankar P., Kumari T., Mohanan P. (2012). Glutaraldehyde treatment elicits toxic response compared to decellularization in bovine pericardium. *Toxicology International*.

[35] Fürst W., Banerjee A. (2005). Release of glutaraldehyde from an albumin-glutaraldehyde tissue adhesive causes significant in vitro and in vivo toxicity. *The Annals of Thoracic Surgery*.

[36] Ho S. T. B., Hutmacher D. W., Ekaputra A. K., Hitendra D., Hui J. H. (2010). The evaluation of a biphasic osteochondral implant coupled with an electrospun membrane in a large animal model. *Tissue Engineering Part A*.

[37] Brehm W., Aklin B., Yamashita T. (2006). Repair of superficial osteochondral defects with an autologous scaffold-free cartilage construct in a caprine model: implantation method and short-term results. *Osteoarthritis and Cartilage*.

[38] Mainil-Varlet P., Rieser F., Grogan S., Mueller W., Saager C., Jakob R. P. (2001). Articular cartilage repair using a tissue-engineered cartilage-like implant: an animal study. *Osteoarthritis and Cartilage*.

[39] Shao X., Goh J. C. H., Hutmacher D. W., Lee E. H., Zigang G. (2006). Repair of large articular osteochondral defects using hybrid scaffolds and bone marrow-derived mesenchymal stem cells in a rabbit model. *Tissue Engineering*.

[40] Schlichting K., Schell H., Kleemann R. U. (2008). Influence of scaffold stiffness on subchondral bone and subsequent cartilage regeneration in an ovine model of osteochondral defect healing. *The American Journal of Sports Medicine*.

[41] Moran C. J., Ramesh A., Brama P. A. J., O’Byrne J. M., O’Brien F. J., Levingstone T. J. (2016). The benefits and limitations of animal models for translational research in cartilage repair. *Journal of Experimental Orthopaedics*.

[42] Vasara A. I., Hyttinen M. M, Lammi M. J (2004). Subchondral bone reaction associated with chondral defect and attempted cartilage repair in goats. *Calcified Tissue International*.

[43] Bothe F., Deubel A.-K., Hesse E. (2019). Treatment of focal cartilage defects in minipigs with zonal chondrocyte/mesenchymal progenitor cell constructs. *International Journal of Molecular Sciences*.

[44] Sciarretta F. V. (2013). 5 to 8 years follow-up of knee chondral defects treated by PVA-H hydrogel implants. *European Review for Medical and Pharmacological Sciences*.

[45] Li G., Yin J., Gao J. (2013). Subchondral bone in osteoarthritis: insight into risk factors and microstructural changes. *Arthritis Research and Therapy*.

