# Supplementary material for: Preclinical Testing of New Hydrogel Materials for Cartilage Repair: Overcoming Fixation Issues in a Large Animal Model
Source: Int J Biomater. 2021 Jun 19;2021:5583815. doi: 10.1155/2021/5583815 (PMC8235960; doi:10.1155/2021/5583815)
Supplement: Supplementary Materials — Supplemental Figure 1: characterisation of the used PCL enforcement. Supplemental Figure 2: in vitro pullout strength comparison of fibrin glue and BioGlue® for CCT-reinforced PAGE hydrogels. Supplemental Table 1: modified O'Driscoll score as used for semiquantitative histomorphological evaluation. [file 5583815.f1.docx]

## Supplementary Materials


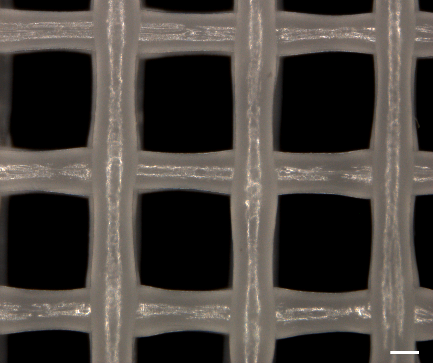

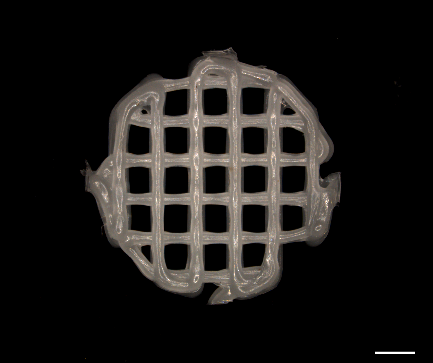

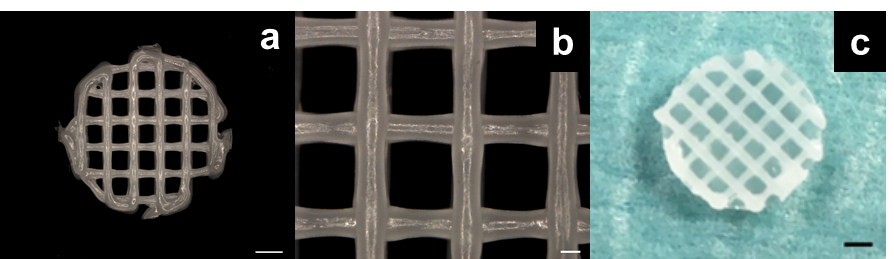
Supplemental Figure 1 Characterisation of the used PCL-enforcement. Overview images of the PCL constructs, scale bar: 1000 µm (a) and magnification of the PCL structure, scale bar: 200 µm (b) The PCL enforcement was soaked with in total 30 µL cell-containing starPEG hydrogel, scale bar: 1000µm (c).

**a**

**c**

**b**

*


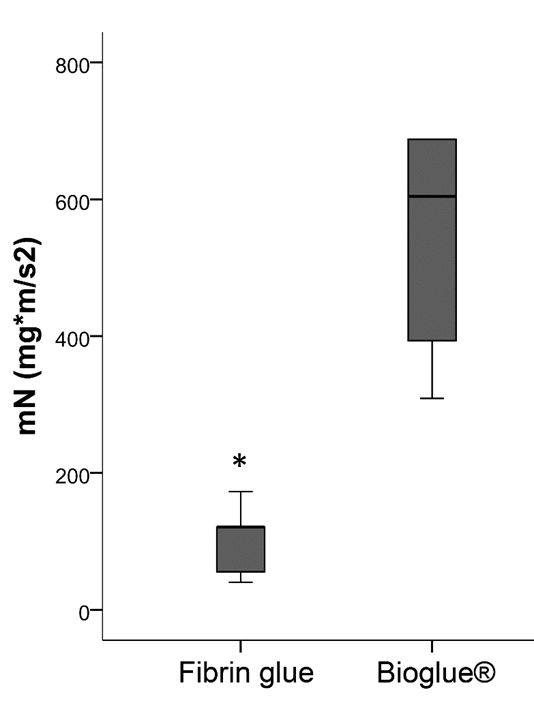


Supplemental Figure 2 In vitro pullout strength comparison of fibrin glue and BioGlue® for CCT-reinforced PAGE-Hydrogels; n=5; Mean pullout strength ± SD per group. *: p ≤ 0.05 vs. BioGlue® (ANOVA)

Supplemental Table 1 Modified O’Driscoll Score as used for semiquantitative histomorphological evaluation.

| **Characteristic** | **Grading** | **Score** |
| --- | --- | --- |
| Degree of defect repair | In level with surrounding cartilage   75% repair of defect depth   50% repair of defect depth   25% repair of defect depth   0% repair of defect depth | 4  3  2  1  0 |
| Integration to border zone | Complete integration with surrounding cartilage   Demarcating border < 1 mm   3/4th of graft integrated, 1/4th with a notable border > 1 mm wide  1/2 of graft integrated with surrounding cartilage, 1/2 with a notable border > 1 mm wide   From no contact to 1/4th of graft integrated with surrounding cartilage | 4  3  2  1  0 |
| Macroscopic appearance | Intact smooth surface   Fibrillated surface   Small, scattered fissures or cracks   Several, small or few but large fissures   Total degeneration of grafted area | 4  3  2  1  0 |
| Total |  | 12 |
